# Zinc Oxide Nanoparticles Damage Tobacco BY-2 Cells by Oxidative Stress Followed by Processes of Autophagy and Programmed Cell Death

**DOI:** 10.3390/nano10061066

**Published:** 2020-05-30

**Authors:** Ľudmila Balážová, Matej Baláž, Petr Babula

**Affiliations:** 1 Department of Pharmacognosy and Botany, The University of Veterinary Medicine and Pharmacy in Košice, Komenského 72, SK-041 81 Košice, Slovakia; 2 Department of Mechanochemistry, Institute of Geotechnics, Slovak Academy of Sciences, Watsonova 45, 040 01 Košice, Slovakia; balazm@saske.sk; 3 Department of Physiology, Faculty of Medicine, Masaryk University, Kamenice 5, CZ-625 00 Brno, Czech Republic; petr-babula@email.cz

**Keywords:** BY-2 cells, ZnO nanoparticles, oxidative stress, autophagy, programmed cell death, phytotoxicity

## Abstract

Nanomaterials, including zinc oxide nanoparticles (ZnO NPs), have a great application potential in many fields, such as medicine, the textile industry, electronics, and cosmetics. Their impact on the environment must be carefully investigated and specified due to their wide range of application. However, the amount of data on possible negative effects of ZnO NPs on plants at the cellular level are still insufficient. Thus, we focused on the effect of ZnO NPs on tobacco BY-2 cells, i.e., a widely accepted plant cell model. Adverse effects of ZnO NPs on both growth and biochemical parameters were observed. In addition, reactive oxygen and nitrogen species visualizations confirmed that ZnO NPs may induce oxidative stress. All these changes were associated with the lipid peroxidation and changes in the plasma membrane integrity, which together with endoplasmatic reticulum and mitochondrial dysfunction led to autophagy and programmed cell death. The present study demonstrates that the phytotoxic effect of ZnO NPs on the BY-2 cells is very complex and needs further investigation.

## 1. Introduction

Nanoparticles (NPs) are structures with at least one dimension of 100 nanometers or less. Their small size facilitates new and often unique properties compared with bulk materials of the same composition [1]. Nowadays, consumers are frequently exposed to nanotechnology products in common items such as cosmetics, clothing, textiles, paints, food packing materials, electronics, etc. [2,3,4]. In 2015, more than 1800 consumer products containing engineered NPs were produced by 622 companies in 32 countries [5]. Their use increases contamination of the environment and they can potentially enter the food chain [6,7,8]. To date, various nanotoxicological studies on different models have been performed. These models have included bacteria, mammalian cells, invertebrates, aquatic organisms, and also plants. Some of these studies indicated the toxicity of NPs [9,10,11,12]. The mechanism of the NP’s adverse effects depends on their chemistry, size and shape [13]. Metallic NPs are able to interact with cellular structures and create reactive oxygen species (ROS). ROS lead to the oxidative stress and are subsequently responsible for the oxidative damage of biomolecules, including lipids and nucleic acids. Damage of biomolecules initiates the cell death [14,15,16]. Epigenetic changes and changes in gene expression have also been recorded [17,18,19]. On the other hand, all these effects have been also reported in free metallic ions. However, the toxicity of NPs differs from the toxicity of free ions. The same concentrations of ions and ions released from the NPs surface do not have the same effects. This fact points to a complex mechanism of the NPs toxicity, where NPs usually yield a higher toxicity than free ions. This also means that the adverse effect of NPs does not result just from the solubilization of ions from the NPs surface [13,20,21,22,23].

Zinc oxide nanoparticles (ZnO NPs) are the second most used metallic oxide NPs (after TiO_2_ NPs). They are present in many materials, e.g., transparent UV-protection films, paints, pigments, facade rating materials, gas sensors, cement, ceramics, and various personal care products [2]. The growing use of ZnO NP-based products increases their occurrence in the environment and, therefore, the potential health risk [24]. There are many studies on the toxicity of ZnO NPs; however, most of them employed animal cell models. Results imply that the ZnO NPs toxicity mechanisms stem from their ability to create ROS [25,26]. On the other hand, the ZnO NPs toxicity is closely connected with changes in intracellular calcium ions and the interactions with biomembranes and some organelles, mainly mitochondria, were also reported [25,27]. ZnO NPs induced genotoxicity which is connected with changes in DNA, chromosomal aberration and structural changes of nucleus [28]. Zinc oxide nanoparticles in an aqueous solution under UV radiation have a phototoxic effect based on the production of H_2_O_2_ and O^2−^ reactive oxygen species. Adverse effects of ZnO NPs have been demonstrated on several plant species including pea, corn, cucumber, rye, zucchini, soybean, wheat, *Fagopyrum esculentum*, *Arabidopsis thaliana*, *Vicia faba*, and *Salicornia* [29,30,31,32,33,34,35,36,37]. Biological effects of ZnO NPs depend on different factors, such as particle size, morphology, surface modification, photocatalytic activity, concentration, plant species, and growth conditions [38]. They involve at least three different mechanisms [39]. Firstly, the release of zinc ions from the NPs surface (solubilization) can lead to an imbalanced zinc homeostasis within the cells. Secondly, surface interactions with different structures potentially involved in the formation of toxic substances (e.g., ROS) can occur [1,40]. The third mechanism results from direct interactions of nanoparticles with biological systems and from the disruption of target structures, e.g., inhibition of photosynthetic activity or disruption of nutrient- and water-transport pathways [39,41,42,43,44]. In conclusion, information about the effect of ZnO NPs on plants at cellular level is still missing. In the light of this fact, we performed a study using *Nicotiana tabacum* L. cv. Bright Yellow-2 suspension-cultured cells (BY-2) as the model system. There are many studies that use the BY-2 cells to evaluate toxic effects of heavy metals, different types of chemicals, pharmaceuticals and also different types of nanoparticles [45,46,47,48]. One of the most recent works used BY-2 cells to evaluate the phytotoxicity of naphthoquinones, mainly in connection with reactive oxygen species and changes in DNA methylation [49]. BY-2 cells are also easily cultivated in laboratory. Their rapid reproduction and the homogeneity of the cell population are favorable factors for their use in nanophytotoxicological studies [50].

The main goal of this work was to evaluate the effect and toxicity of commercially available ZnO NPs on the BY-2 cells model to determine possible mechanisms of ZnO NPs toxicity.

## 2. Materials and Methods

### 2.1. Chemicals

All chemicals were obtained from Sigma-Aldrich, St. Louis, MO, USA unless otherwise noted. We have used the same ZnO NPs (approximate crystallite size 46 nm and specific surface area 26 m^2^/g) which have been characterized in our previous work [33]. Murashige and Skoog cultivation medium (MS) including macroelements, microelements, and vitamins was purchased from Duchefa Biochemie B.V., Haarlem, The Netherlands. All fluorescence probes were obtained from Life Technologies, Carlsbad, CA, USA. They were stored in compliance with the manufacturer’s recommendations. Working solutions and all fluorescence probes were prepared immediately before use and handled in compliance with the manufacturer’s instructions.

### 2.2. Cultivation of the BY-2 Cell Suspension

*Nicotiana tabacum* L. cv. Bright Yellow-2 suspension-cultured cells were obtained from Mendel University in Brno, Brno, Czech Republic. The cell culture was well-established at Department of Natural Drugs, University of Veterinary and Pharmaceutical Sciences Brno. Cells were grown in liquid MS medium modified by Nagata [43] supplemented with sucrose (30 g/L), thiamine (1 mg/L), KH_2_PO_4_ (0.2 g/L), and 2,4-dichlorophenoxyacetic acid (0.2 mg/L) under constant shaking at 135 rpm (Kuhner Shaker, type: LT-W, Adolf Kuhner AG, Birsfelden, Switzerland), 27 ± 1 °C in the dark in 250 mL Erlenmeyer flasks. Cells in the exponential phase of growth were exposed to ZnO NPs (particle size <50 nm, Sigma–Aldrich, St. Louis, MO, USA) added into the cultivation medium in concentrations 0, 10, 100, and 400 mg/L, respectively. The treatment and the control samples were collected under sterile conditions at 0, 24, 48, and 72 h, respectively.

### 2.3. Cell Viability and Growth

The viability of BY-2 cells was determined by modified double staining methods using fluorescein diacetate (FDA) and propidium iodide (PI) according to Babula et al. [51]. A fluorescence microscope (Axioskop 40, Carl Zeiss, Göttingen, Germany) equipped with an appropriate set of filters was used. Growth parameters were examined as the packed cell volume (PCV) and fresh weight (FW) according to Krystofova et al. [45].

### 2.4. Spectrophotometric Measurements

#### 2.4.1. Dehydrogenase and Oxidoreductase Activity and the Loss of the Plasma Membrane Integrity

The TTC (2,3,5-triphenyltetrazolium chloride) assay, MTT (methylthiazolyldiphenyl-tetrazolium bromide) assay and the loss of the plasma membrane integrity evaluated by accumulation of Ewans blue (0.05% *w*/*v*) were used in separate assays as described by Poborilova et al. [39]. Dehydrogenase and oxidoreductase activities and the loss of the plasma membrane activity were each expressed as a percentage of the control sample at the beginning of experiment (=100%).

#### 2.4.2. Determination of Caspase-Like, Protease and Acid Phosphatase Activities

Caspase-like activity was evaluated using the commercial Caspase 3 colorimetric kit on the Helios Epsilon Unicam spectrophotometer (ThermoFisher Scientific, Waltham, MA, USA).

Protease activity was determined according to a procedure described by Vagnerova and Macura employing azocasein as a substrate [52], with some modifications noted in [53].

To determine the acid phosphatase activity, an acid phosphatase assay kit using p-nitrophenyl phosphate as a substrate was used in compliance with the manufacturer’s instructions. Results were normalized to protein levels of cell lysates obtained from the same samples and expressed as a percentage of the activity determined for a control sample at the beginning of the experiment (=100%).

#### 2.4.3. Quantification of Total Phenolics and PAL Activity

The amount of soluble phenolics was measured using the Folin–Ciocalteu method; gallic acid was used as a standard [46].

Phenylalanine ammonia-lyase (PAL, EC 4.3.1.24) activity was studied according to Ferrarese et al. [54], and consequently measured according to dos Santos et al. [55], with slight modifications noted in [56]. A Cytation 3 system multi-mode reader (BioTek, Winooski, VT, USA) was used for the spectrophotometry. PAL activity was expressed in nmol·min^−1^·mg^−1^ protein; content of proteins was determined according to Bradford [57].

### 2.5. Extraction and Quantification of Proteins, In-Gel Assays, and Western Blot

#### 2.5.1. Extraction and Quantification of Proteins

Samples were ground with a mortar and pestle in liquid nitrogen, where sea sand and PVPP were added directly to the mortar for each sample. Extraction buffer containing 250 mM Tris-HCl (pH 8.48), sucrose, EDTA (ethylenediaminetetraacetic acid), L-cysteine, reduced glutathione and a protein inhibitor cocktail (phenylmethylsulfonyl fluorid, aprotinin, and pepstatin) was added into the sample in a 1:5 ratio (*w*/*v*), as described by Brychkova et al. [58]. The content of total soluble proteins was determined according to Bradford [57].

#### 2.5.2. In-Gel Assays

Protein extracts were fractionated by native gel electrophoresis with 10% (*w*/*v*) separating gel and 4% (*w*/*v*) stacking gel, in the absence of sodium dodecyl sulfate, under non-denaturing conditions. The gels were run at 40 mA for 1–3 h in a cooled (4 °C) system (Mini-PROTEAN1II, Bio-Rad, Hercules, CA, USA). In-gel assays of peroxidase (PER, EC 1.11.1.7), superoxide dismutase (SOD, EC 1.15.1.1), and ascorbate peroxidase (APER, EC 1.11.1.11) activities were examined after fractionation of proteins in native gel. For SOD activity, a modified assay described by Brewer was used [59]. The gels were immersed in 0.1 M Tris–HCl (pH 7.5) supplemented with 0.1 mM phenazine methosulfate and 1 mM MTT, and incubated at room temperature, with gentle shaking. A reaction mixture containing 0.4 mg/mL MTT, 0.2 mg/mL phenylmethylsulfonyl fluoride and 0.2 mg/mL MgCl_2_ in 20 mL of 50 mM Tris-HCl buffer (pH 8.5) was used to evaluate APER activity as described by Mittler et al. [60]. In the modified assay for PER described by Gregory et al. [61], H_2_O_2_ serves as a proton-accepting substrate while 10 mg/mL diaminobenzidine (DAB) in 50 mM phosphate buffered saline (PBS) (pH 7.0) serves as a proton-donor substrate. The in-gel detected bands were scanned by an Arcus 1200 scanner. Relative intensities of the bands were estimated by the software Image J 1.48 (NIH Image, Madison, WI, USA). All results were normalized against protein concentrations of cell lysates taken from the same samples.

#### 2.5.3. Western Blot

The amount of glutathione reductase (GR, EC 1.6.4.2) in BY-2 cells was determined by the Western blot analysis. Protein extracts (50 µg) were separated by SDS-PAGE on 10% (*w*/*v*) polyacrylamide separating gel and 4% (*w*/*v*) stacking gel at 10 mA, as described by Sagi et al. [62]. The gels were blotted onto PVDP (Millipore, Bedford, MA, USA) according to Brychkova et al. [63] The immunodetection was carried out with the polyclonal antibody anti-GR (Agrisera, Vännäs, Sweden), followed by the horseradish-conjugated secondary antibody (anti-rabbit IgG-HSP, Sigma-Aldrich, St. Louis, MO, USA). Protein bands were visualized using Gel-Doc (Bio-Rad, Hercules, CA, USA) and SuperSignal West Pico Chemiluminescent Substrate (ThermoFisher Scientific, Waltham, MA, USA). Densitometry was used to quantify amount of enzymes (software Image J 1.48, NIH Image, Madison, WI, USA).

### 2.6. Microscope Observations

#### 2.6.1. Visualization of the Cell Structure—Endoplasmic Reticulum, Golgi Apparatus, and Mitochondria

Changes in the structure and stress of endoplasmic reticulum were observed using a fluorescence probe ER Tracker Red (587/615 nm; 0.5 µM in MS supplemented with 0.5 mM calcium sulphate), a cell-permeant, live-cell stain that is highly selective for the endoplasmic reticulum (ER). Mitochondria were visualized using two fluorescence probes, MitoTracker Green FM (490/516 nm, Life Technologies, Carlsbad, CA, USA), a green-fluorescent mitochondrial stain, which appears to localize mitochondria regardless of the mitochondrial membrane potential, and MitoRed (569/594 nm), a probe, which stains mitochondria according to the mitochondrial potential. The BY-2 cells were washed three times with fresh MS and incubated (final concentrations—200 µM for MitoTracker Green FM and 150 nM for MitoRed). BODIPY^®^ TR Ceramide (589/617 nm, Life Technologies, Carlsbad, CA, USA) was used to visualize the Golgi apparatus (GA). All the visualizations were performed according to manufacturer’s instructions. A confocal microscope Leica TCS SP8 X (Leica, Wetzlar, Germany) and appropriate excitation/emission wavelengths were used for all observations. The NIS-elements software (Nikon, Minato, Japan) was used to process images and to analyze the resultant pictures.

#### 2.6.2. Visualization of Intracellular ROS and RNS

The production of ROS, especially of hydroxyl radicals, was determined by 2´,7´-dichlorodihydrofluorescein diacetate (H_2_DCF DA, 492–495/517–527 nm, Life Technologies, Carlsbad, CA, USA), in compliance with a procedure described by Poborilova et al. [49]. 3′-(p-aminophenyl) fluorescein (APF, 490/515 nm, Life Technologies, Carlsbad, CA, USA) was used to visualize hydroxyl radicals, peroxynitrite and hypochlorite anions. RNS were visualized using 4-amino-5-methylamino-2′,7′-difluorofluorescein diacetate (DAF-FM DA, 495/515 nm). Samples were examined using the confocal microscope Leica TCS SP8 X (Leica, Wetzlar, Germany), at appropriate excitation/emission wavelengths. The NIS-elements software (Nikon, Minato, Japan) was used to process images and to evaluate the resultant pictures.

#### 2.6.3. Visualization of Zinc Ions

RhodZinTm-3 AM (550/575 nm, Life Technologies, Carlsbad, CA, USA), a cell-permeant ester, was used to visualize intracellular free zinc ions. BY-2 cells were washed three times with PBS buffer (0.1 M, pH 7.4) and incubated with the probe (final concentration 10 µM, 1 h, 23 ± 1 °C, darkness). After incubation, the cells were examined with the confocal microscope Leica TCS SP8 X (Leica, Wetzlar, Germany), at appropriate excitation/emission wavelengths. The NIS-elements software (Nikon, Minato, Japan) was used to process images and to evaluate the resultant pictures.

#### 2.6.4. Mitotic Index, Nuclear Architecture, and DNA Fragmentation

This procedure was used in order to observe nuclei and to determine mitotic index as well as DNA fragmentation. Nuclear architecture and mitotic index was examined with a fluorescent probe Hoechst 33258. Approximately 1000 nuclei were observed in each preparation using a fluorescence microscope (Olympus AX 70, Hamburg, Germany) equipped with broad-spectrum UV excitation. Every morphological change as well as the mitotic index was expressed as a percentage of the total cells. A terminal deoxynucleotidyl transferase (Tdt)-mediated deoxyuridinetriphosphate (dUTP)-nick labelling (TUNEL) assay was performed using a TMR-red in situ cell death detection kit (Roche, Basel, Switzerland), in compliance with the manufacturer’s instructions, as described by Poborilova et al. [46]. Approximately 1000 cells per sampling time per treatment were counted in triplicate; the amount of positive BY-2 cells was expressed in percentages.

### 2.7. Statistical Analyses

GraphPad Prism 5 (GraphPad, San Diego, CA, USA) was used for statistical analyses. ANOVA was used to evaluate the data. The results were expressed as the mean ± S.E.M. Values of *p* < 0.05 (*), *p* < 0.01 (**), and *p* < 0.001 (***) were considered significant.

## 3. Results

### 3.1. The Effect of ZnO NPs on the Viability and Cell Growth

**Figure 1:**
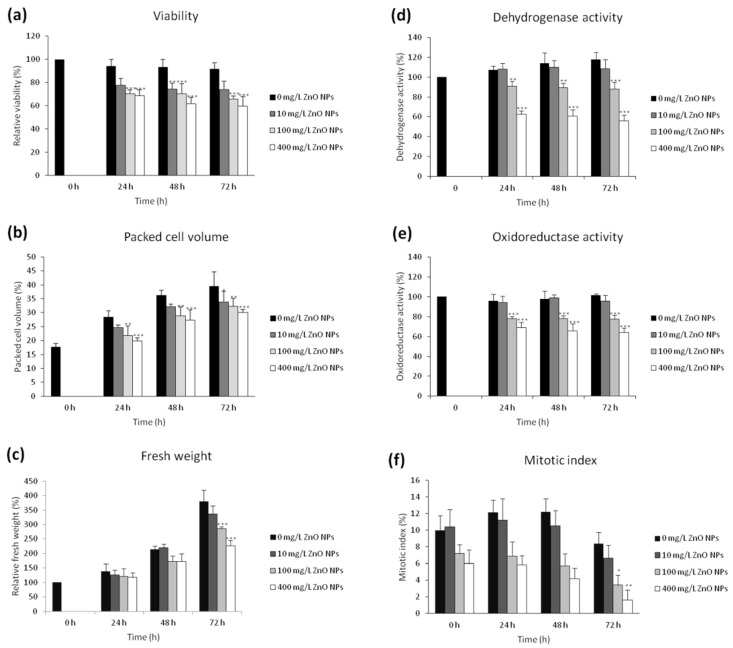
The effect of ZnO NPs on viability (**a**), packed cell volume (**b**), fresh weight (**c**), dehydrogenase activity (**d**), oxidoreductase activity (**e**) and mitotic index (**f**) of tobacco BY-2 cells. Cells were harvested at strictly defined time intervals (0, 24, 48, and 72 h) after the treatment. Each point represents the mean of three independent experiments. * indicates a significant difference between BY-2 cells treated with ZnO NPs and the corresponding control (untreated) BY-2 cells at *p* < 0.05, ** indicates a significant difference between the BY-2 cells treated with ZnO NPs and the corresponding control (untreated) BY-2 cells at *p* < 0.01, *** indicates a significant difference between the BY-2 cells treated with ZnO NPs and the corresponding control (untreated) BY-2 cells at *p* < 0.001. Bars represent standard deviations.

The viability of BY-2 cells after the ZnO NPs treatment was examined by fluorescence double staining using FDA and PI. The viability of the control remained high (94.7 ± 3.6%) during the whole cultivation. A significanty decreased viability (*p* < 0.001) was already observed 24 h after the exposure to ZnO NPs in the two highest concentrations, 100 and 400 mg/L, respectively. The most significant reduction in viability (*p* < 0.001) was observed in 400 mg/L after 72 h (59.6 ± 8.2%). The lowest concentration of ZnO NPs (10 mg/L) had no significant effect on the viability during the entire experiment. The viability decreased significantly (*p* < 0.001) in both time- and dose-dependent manners in 100 and 400 mg/L, respectively (Figure 1a). Furthermore, we also evaluated the effect of free zinc ions on the viability using the same concentrations. In this case, a decreased viability was observed in two concentrations, 100 and 400 mg/L, respectively. Nevertheless, the viability was higher than after the ZnO NPs exposure. In the case of 400 mg/L after 72 h, the viability was 59.6 ± 8.2% for ZnO NPs and 67.5 ± 4.7% for zinc ions. The used chemical forms of Zn induced cell death to different extents in a dose-dependent fashion. Relative efficacy of different chemical forms of zinc on the viability was higher for ZnO NPs than in the case of zinc ions.

To determine the effect of ZnO NPs on BY-2 cells growth parameters, PCV, FW, and mitotic index were probed. ZnO NPs in concentrations 100 mg/L and 400 mg/L had a significant (*p* < 0.01 or *p* < 0.001) negative impact on the growth measured as PCV. PCV after 24 h for the control, 100 mg/L and 400 mg/L were 28.4 ± 2.3%, 21.8 ± 3.4%, and 19.9 ± 1.0%, respectively. At the end of the experiment (72 h), PCV for the control, 100 and 400 mg/L were 39.4 ± 5.1%, 32.3 ± 2.8% and 30.1 ± 1.1%, respectively (Figure 1b). These results corresponded with FW values. The highest concentration of ZnO NPs (400 mg/L) led to a significant (*p* < 0.001) reduction of FW to 226.4 ± 18.8% after 72 h (FW of the control was 380.1 ± 39.0%). The concentration of 100 mg/L ZnO NPs significantly (*p* < 0.001) reduced the growth as well (285.2 ± 8.3%) (Figure 1c). When compared with ZnO NPs, PCV values for free zinc ions after 72 h were as follows: 35.2 ± 2.3% for 100 mg/L and 33.9 ± 1.7% for 400 mg/L. FW for 100 mg/L was 324.4 ± 15.5% and 283.1 ± 19.2% was evidenced for 400 mg/L concentration of ZnO NPs.

Determination of dehydrogenase (MTT assay) and oxidoreductase (TTC assay) activities is generally used to evaluate the cell metabolic activity, which is proportional to the number of viable cells. A significantly decreased activity of MTT (*p* < 0.01) and TTC (*p* < 0.001) was already observed after 24 h of exposure to ZnO NPs. Results showed a gradual decrease in the activity of both enzymes in the two highest concentrations of ZnO NPs (100 and 400 mg/L, respectively). At the end of the experiment (72 h), the dehydrogenase activity was 88.0 ± 6.6% and 56.1 ± 5.9% for 100 and 400 mg/L ZnO NPs, respectively. The oxidoreductase activity was 77.4 ± 4.0% and 64.1 ± 4.7% for 100 and 400 mg/L ZnO NPs, respectively (Figure 1d,e).

A significantly (*p* < 0.05 or *p* < 0.01) decreased mitotic index was only found in 100 and 400 mg/L, after 72 h. While the mitotic index of the control was 8.37 ± 1.40%, it dropped to 3.43 ± 1.19% in 100 mg/L ZnO NPs and to 1.63 ± 1.21% after the exposure to 400 mg/L ZnO NPs. At the onset of the experiment, the mitotic index was 9.97 ± 1.81%. All values indicate the ability of ZnO NPs to reduce the growth in a concentration-dependent manner. The most significant reduction was observed at the end of the experiment (Figure 1f).

### 3.2. Visualization and Quantification of Intracellular Zinc Ions, ROS and RNS in Connection with Changes in the BY-2 Cell Structure

**Figure 2:**
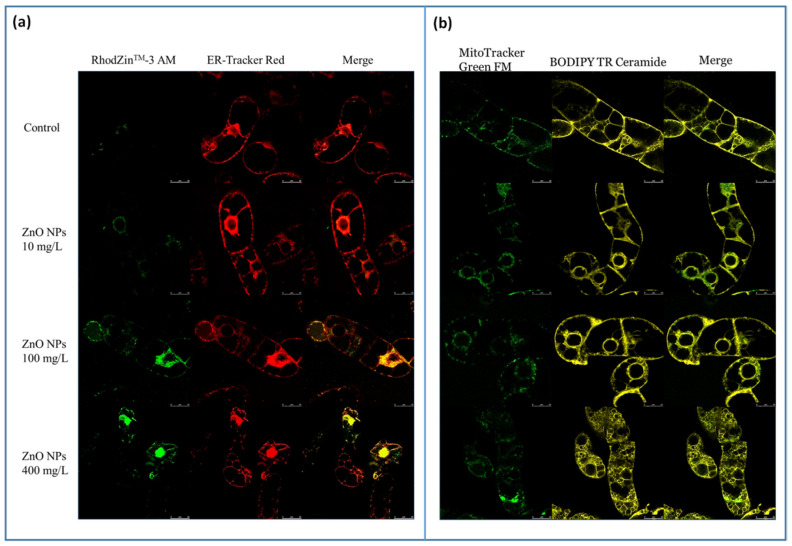
The effect of 0, 10, 100 and 400 mg/L ZnO NPs on BY-2 cells 72 h after the treatment. Visualization of intracellular zinc ions using RhodZinTM-3 AM (**a**). Endoplasmic reticulum was visualized by ER-Tracker Red (**a**), mitochondria by MitoTracker Green FM, and Golgi apparatus by BODIPY^®^ TR Ceramide (**b**). The length of bar represents 25 µm.

The presence of ZnO NPs led to an increase in free intracellular zinc ions in a concentration-dependent manner (Figure 2a). Free zinc ions were mainly observed in the cell wall (already after 24 h of treatment), around nuclei and in cytoplasm adjacent to the cell wall. Free zinc ions were also observed in nuclei in the case of the concentration 400 mg/L. Colocalization with endoplasmic reticulum (ER) showed that free zinc ions are partially accumulated in this structure (mainly around nuclei).

Morphological changes in the ER were also observed. In the control, ER was mainly located around nuclei, with weak distribution also in cytoplasm adjacent to the cell wall, in the case of 400 mg/L ZnO NPs, ER created a complex network, predominantly around nuclei.

**Figure 3:**
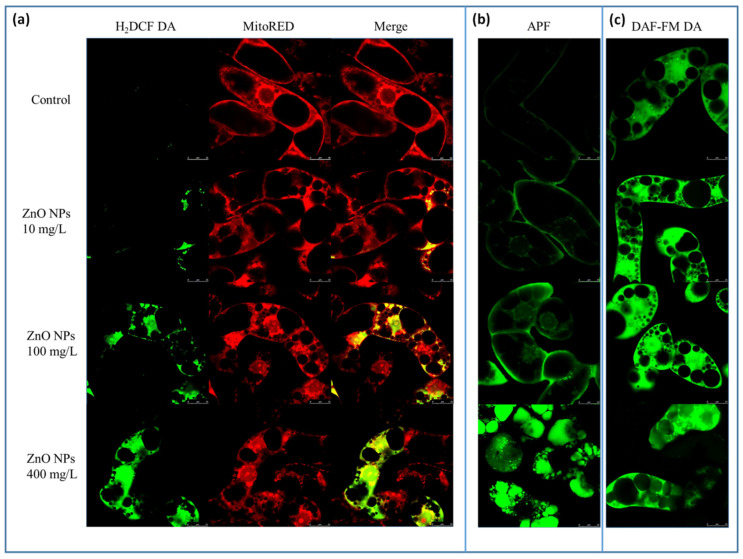
The effect of 0, 10, 100, and 400 mg/L ZnO NPs on BY-2 cells 72 h after the onset of the treatment. Visualization of ROS, mainly hydroxyl radicals, by 2´,7´-dichlorodihydrofluorescein (H2DCF DA), mitochondria according to its potential by MitoRED (**a**), hydroxyl radicals, peroxynitrite, and hypochlorite anions using 3′-(p-aminophenyl) fluorescein (APF) (**b**); reactive nitrogen species visualized by 4-amino-5-methylamino-2′,7′-difluorofluorescein diacetate DAF-FM DA (**c**). The length of bars represents 25 µm.

MitoTracker Green FM is relatively insensitive to mitochondrial membrane potential and oxidative stress, therefore it can be used to evaluate the mitochondrial distribution and morphology regardless of these parameters. In the control, mitochondria were distributed regularly in the cytoplasm. In 400 mg/L ZnO NPs, a formation of fluorescent aggregates around nuclei was observed. In addition, a small amount of aggregates was also observed in cytoplasm adjacent to plasma membrane. The presence of aggregates probably indicates a mitochondrial dysfunction. The formation of aggregates was also observed when MitoRed probe was used (Figure 3a). The mitochondrial dysfunction is also supported by formation of strongly fluorescent red aggregates around nuclei with an increasing amount of ZnO NPs, and a loss of emission in other parts of cytoplasm, mainly adjacent to the cell wall, were well evident. In this part of cytoplasm, only small emitting spots were observed.

Golgi apparatus (GA) exhibited the most significant morphological changes. Whereas the control showed a regular distribution of GA in cytoplasm, the application of ZnO NPs led to morphological changes including the accumulation of small vesicular structures around nuclei and in cytoplasm adjacent to the cell wall. The concentration 400 mg/L caused a rearrangement of GA into a complex network (Figure 2b), which corresponded to a strong vacuolization. The presence of many small vacuoles was well evident when using APF and DAF-FM DA staining.

ROS were visualized using two probes—general marker of oxidative stress H2DCF DA and APF, hydroxyl radicals, hypochlorite, and a peroxynitrite sensor (Figure 3a,b). Both probes revealed the ability of ZnO NPs to generate ROS in a concentration-dependent manner. In the control, the presence of ROS was only observed in the cytoplasm adjacent to the cell wall after 72 h. However, the intensity of the signal was very low. The concentration of 10 mg/L ZnO NPs generated ROS in cytoplasm around nuclei but not in nuclei. Higher concentrations increased the generation of ROS in cytoplasm. For 100 mg/L ZnO NPs, ROS were visible around nuclei with weak signal in nuclei and nucleoli and partially in cytoplasm adjacent to the cell wall. Colocalization with MitoRed revealed that these spots corresponded to mitochondria, especially to mitochondrial aggregates. The concentration of 400 mg/L ZnO NPs showed a very strong fluorescence of both cytoplasm and nuclei. Comparatively, the APF probe revealed the presence of hydroxyl radicals, hypochlorite and peroxynitrite in cytoplasm mainly around nuclei in 10 mg/L ZnO NPs, which corresponds to localization of mitochondria. The concentration of 100 mg/L ZnO NPs showed a fluorescence signal in cytoplasm adjacent to cell wall, in cytoplasmic strands and around nuclei. While nuclei showed no signal, the signal of nucleoli was well evident in this case. The application of 400 mg/L ZnO NPs led to a very strong production of ROS in all parts including vacuoles. Whereas the fluorescence signal of vacuolar content was relatively weak in older cells with several large vacuoles, individual small vacuoles showed a very strong fluorescence signal in cells with strong vacuolization.

The determination of RNS in the cells following the treatment with different concentrations of ZnO NPs indicated a significant dose-dependent increase in their formation. Differences between the two highest concentrations of ZnO NPs were observed. While 100 mg/L ZnO NPs showed a very strong fluorescence in almost all cell parts including nuclei and nucleoli (except vacuoles), 400 mg/L NPs showed almost no fluorescence in nuclei and nucleoli but medium fluorescence of the vacuolar content. However, fluorescence intensities were almost identical (Figure 3c).

**Figure 4:**
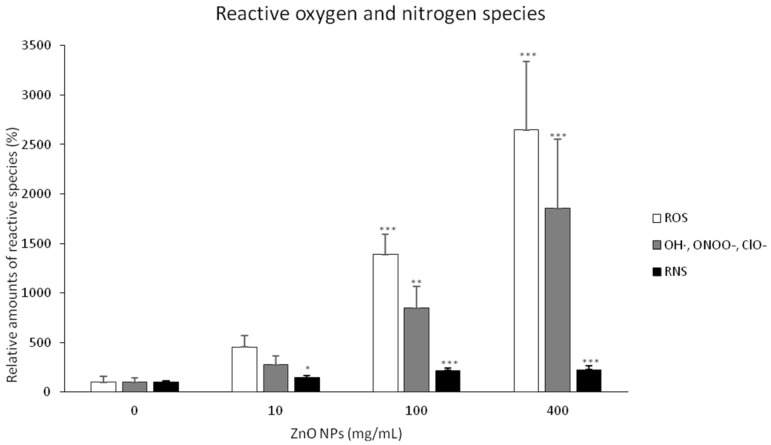
Formation of reactive oxygen and nitrogen species induced by ZnO NPs 72hrs after the treatment. Changes in the amount of ROS were expressed as hydroxyl (white column), hydroxyl radicals, peroxynitrite, and hypochlorite anions (grey column), and reactive nitrogen species as nitric oxide (black column). Each bar represents the mean of three independent experiments. * indicates a significant difference between BY-2 cells treated with ZnO NPs and the corresponding control (untreated) BY-2 cells at *p* < 0.05, ** indicates a significant difference between BY-2 cells treated with ZnO NPs and the corresponding control (untreated) BY-2 cells at *p* < 0.01, *** indicates a significant difference between BY-2 cells treated with ZnO NPs and the corresponding control (untreated) BY-2 cells at *p* < 0.001. Bars represent standard deviations.

To quantify changes in the production of ROS and RNS, we used the image processing and analysis (Figure 4). A non-significant increase in the production of ROS was recorded for the lowest concentration of ZnO NPs. The amount of ROS and RNS was significantly increased for both 100 and 400 mg/L ZnO NPs. However, the differences in RNS production for the plants exposed to these two highest concentrations of ZnO NPs were negligible. These results imply the ability of ZnO NPS to induce oxidative stress and subsequently morphological changes. The colocalization revealed the places of formation of ROS—mitochondria, nucleus, nucleolus, and vacuoles (Figure 3).

### 3.3. Changes in Biochemical Parameters—Antioxidant Enzymes

The function of the antioxidant enzyme system was evaluated in order to understand the mechanism of ZnO NPs, and especially their ability to generate ROS.

ZnO NPs showed an ability to decrease the SOD activity in all applied concentrations (*p* < 0.01 or *p* < 0.001) (Figure 51a). The activity of SOD isozymes in BY-2 cells was found to be time-dependent. The SOD activity determined after 24 h was 69.5 ± 3.3% and after 72 h 31.7 ± 7.4%. We can observe a time-dependent reduction of SOD by ZnO NPs in connection with increased amounts of ROS and RNS, as determined after 72 h.

A significantly (*p* < 0.001) reduced activities of PER were observed after 24 h in 10 and 100 mg/L ZnO NPs; 400 mg/L ZnO NPs showed an activity comparable with the control. The decreased PER activity was observed after 48 h in almost all experimental variants (*p* < 0.001) except 400 mg/L ZnO NPs, where the activity was comparable with the control. On the other hand, significant (*p* < 0.001) differences were observed after 72 h of treatment. While 10 mg/L showed a significantly increased PER activity (120.2 ± 4.3%), 100 and 400 mg/L ZnO NPs showed a significant reduction (63.6 ± 1.3% and 27.5 ± 2.2%) (Figure 51b).

We evaluated changes in the activity of two isoforms APER1 and APER2 after 72 h. Activities of both APER isozymes gradually decreased (*p* < 0.001) in all experimental variants with the increasing amount of ZnO NPs. The relative activity values in 400 mg/L ZnO NPs were 70.0 ± 2.9% for APER1 (Figure 52a) and 58.3 ± 2.0% for APER2, respectively (Figure 52b). Differences in both APER1 and APER2 activities for 10 and 100 mg/L ZnO NPs were negligible, but were lower than that of control.

GR catalyzes the reduction of oxidized glutathione (GSSG) to its reduced form (GSH). Reduced glutathione is a key intracellular scavenger of reactive oxygen species. While SOD, PER, and APER showed significantly reduced activities in 400 mg/L ZnO NPs, the amount of GR was significantly (*p* < 0.001) higher in all experimental variants in a concentration-dependent manner after 72 h (Figure 52c).

### 3.4. Changes in Total Phenolics and PAL

**Figure 6:**
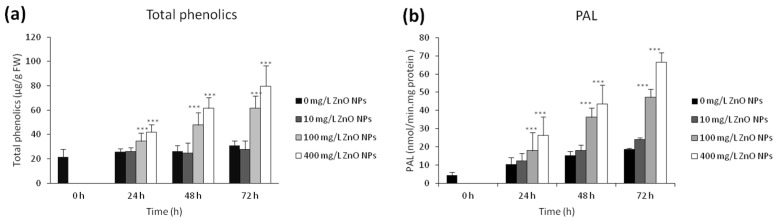
Content of total polyphenols (µg/g FW) (**a**) and phenylalanine ammonia-lyase (PAL) (**b**) activity (pcat/g FW) in control and BY-2 cell treated with 10, 100, and 400 mg/L ZnO NPs. *** indicates a significant difference between the BY-2 cells treated with ZnO NPs and the corresponding control (untreated) BY-2 cells at *p* < 0.001. Bars represent standard deviations.

Due to the ZnO NPs ability to generate ROS, we focused on the content of phenolics as important antioxidants and protective compounds. The amount of total phenolics was significantly increased upon exposure to ZnO NPs and different amounts were detected when various concentrations of ZnO NPs were used. While the content of phenolics in 10 mg/L ZnO NPs was comparable with the control, the amounts were significantly higher (*p* < 0.001) for 100 and 400 mg/L ZnO NPs in a concentration-dependent manner (31.1 ± 3.7 µg/g FW for control, 61.6 ± 10.0 µg/g FW for 100 mg/L ZnO NPs and 79.7 ± 16.6 µg/g FW for 400 mg/L ZnO NPs, respectively; Figure 6a).

Due to the fact that enzyme phenylalanine ammonia lyase (PAL) is crucial in the biosynthesis of many plant phenolic compounds, we decided to evaluate its activity. We observed that 100 and 400 mg/L ZnO NPs significantly (*p* < 0.001) enhanced the PAL activity in a time-dependent manner. The values of PAL activity were 31.1 ± 3.7 nmol·min^−1^·mg^−1^ protein for control, 47.3 ± 4.4 nmol·min^−1^·mg^−1^ protein for 100 mg/L and 66.6 ± 5.3 nmol·min^−1^·mg^−1^ protein for 400 mg/L ZnO NPs, respectively (Figure 6b). The exposure to 10 mg/L ZnO NPs caused no changes in the PAL activity.

### 3.5. A Loss of the Plasma Membrane Integrity

The oxidative damage of lipids, i.e., lipid peroxidation represents a mechanism of damage to biomembranes. The damage of cell membranes, i.e., the “a loss of the plasma membrane integrity”, was measured as an accumulation of Evans blue stain that only penetrates disrupted or damaged plasma membranes with changed permeability. The uptake of Evans blue stain after 72 h was significantly (*p* < 0.001) higher in all experimental variants. The values of Evans blue accumulation were 127.4 ± 16.5% for 10 mg/L ZnO NPs, 160.9 ± 9.6% for 100 mg/L ZnO NPs, and 182.6 ± 20.4% for 400 mg/L ZnO NPs, respectively. For the details see Figure 71a. The values of the Evans blue accumulation for 100 and 400 mg/L ZnO NPs were significantly higher in both concentration- and time-dependent manners. ZnO NPs demonstrated plasma membrane damage and a loss of the plasma membrane integrity. These results correspond to the previous results, mainly to those showing the ability of ZnO NPs to cause generation of ROS and RNS with a subsequent lipid peroxidation.

### 3.6. Changes in Nuclear Architecture and Ability of ZnO NPs to Induce Programmed Cell Death and Autophagy

In our work, we studied the process of programmed cell death using different approaches including the evaluation of nuclear architecture and changes typical for programmed cell death (PCD) (condensation of chromatin, presence of irregular or stretched nuclei, and presence of the apoptic-like bodies), TUNEL assay for the detection of DNA fragmentation that results from PCD cascade, and caspase-like activity (by using a chromogenic substrate and acetyl-Asp-Glu-Val-Asp-p-nitroanilide). In addition, we determined protease and acid phosphatases activities because these enzymes are crucial for autophagy processes.

PCD in plants is closely connected with the activation of core executive enzymes exhibiting caspase-like activity. A significant increase in the caspase-like activity was detected in all ZnO NPs-treated groups. After 24 h, the highest increase in the caspase-like activity was determined in 100 mg/L ZnO NPs (166.9 ± 12.0%; control = 100%, *p* < 0.001). On the other hand, 10 mg/L ZnO NPs yielded values comparable with the control (111.2 ± 16.3%). After 72 h of the treatment, the caspase-like activity was significantly (*p* < 0.001) increased in all experimental variants; the values were 157.9 ± 9.6% for 10 mg/L ZnO NPs, 183.2 ± 16.6% for 100 mg/L ZnO NPs, and 184.3 ± 10.5% for 400 mg/L ZnO NPs, respectively (Figure 71c).

TUNEL test confirmed the DNA fragmentation, an important sign of PCD. The number of TUNEL-positive cells increased after 48 h in a concentration-dependent manner. The number of the TUNEL-positive cells in the control was very low, 1.7 ± 0.9%, 10 mg/L ZnO NPs showed 2.9 ± 1.5% of TUNEL-positive cells (ns), 100 mg/L ZnO NPs showed 21.9 ± 7.7% (*p* < 0.001) and 400 mg/L ZnO NPs showed 29.2 ± 8.0% (*p* < 0.001) (Figure 72b).

Processes of PCD were well evident and observable during the final step, where the DNA fragmentation and changes in nuclear architecture could be evaluated. Changes in the nuclear architecture were evaluated microscopically as the presence of irregular, stretched and apoptic-like nuclei, condensation of chromatin and the micronuclei (Figure 72a). The most significant changes in nuclear morphology were determined in the BY-2 cells exposed to 400 mg/L ZnO NPs. The number of normal nuclei decreased from 95.4 ± 1.4% for the control to 48.4 ± 3.1% for 400 mg/L ZnO NPs. Whereas no apoptic-like nuclei and micronuclei were observed in the control, their amount in 400 mg/L ZnO NPs was large (6.5 ± 1.2% of cells with apoptic-like bodies and 2.3 ± 1.3% of cells with micronuclei). Nuclei with condensed chromatin were detected in 10.3 ± 2.7% in 400 mg/L (compared with 0.4 ± 0.4% in the control). Stretched nuclei were present in 13.5 ± 4.3% in 400 mg/L (0.9 ± 0.4% in the control). Changes in nuclear architecture were insignificant in the lowest concentration of ZnO NPs. The most significant changes in nuclear morphology were observed when the 400 mg/L ZnO NPs were used.

Acid phosphatase is a marker enzyme of autolysosomes that play a crucial role in autophagy, a cellular self-digestion. ZnO NPs significantly increased the activity of acid phosphatase in all concentrations, with the following values after 72 h: 129.8 ± 11.7% in the control, 192.0 ± 4.0% in 10 mg/L ZnO NPs, 262.4 ± 19.5% in 100 mg/L ZnO NPs, and 314.8 ± 22.1% in 400 mg/L ZnO NPs, respectively (the difference was significant for all ZnO-NPs treated cells, *p* < 0.001) (Figure 71d). These results indicate that ZnO NPs induced autophagy in BY-2 cells.

Another sign of autophagy is the enhanced protease activity. The enhanced protease activity was observed in all ZnO NPs concentrations. The greatest protease activity was determined in 400 mg/L ZnO NPs; the activity was high at all time intervals: 209.7 ± 22.1% after 24 h, 305.0 ± 52.1% after 48 h, and 404.7 ± 41.5 after 72 h, respectively (all *p* < 0.001) (Figure 71b). The concentration of 10 mg/L ZnO NPs showed insignificant changes in the protease activity. These results corresponded to those for acid phosphatase activity.

All methods employed during the experiment to determine PCD (TUNEL assay, caspase-like activity, nuclear morphology) and two assays (acid phosphatase and protease activities) connected with autophagy provided us complex information on the ability of ZnO NPs to initiate the processes of cell death in ZnO NPs-treated BY-2 cells.

## 4. Discussion

The cytotoxic effect of different types of NPs (Al_2_O_3_, γ-Fe_2_O_3_) on BY-2 tobacco cells was studied; however, no study evaluating an effect of ZnO NPs has been published yet. The toxicity of aluminum oxide NPs on BY-2 cells was previously demonstrated [46]. Krystofova et al. reported a moderate effect of magnetic γ-Fe_2_O_3_ nanoparticles on the growth of the BY-2 cell suspension. On the contrary, these authors described changes in some biochemical parameters, such as in the glutathione S-transferase activity and in the content of protein and phytochelatin [45]. Negative effects of ZnO NPs were also studied on wheat callus cells of two varieties—Parabola (stress-tolerant) and Raweta (sensitive) and banana in vitro cultures [64,65]. Our results were in agreement with outcomes of other works that demonstrated the toxicity of NPs to a variety of plants species, e.g., soybeans, *Arabidopsis,* eggplants and *Salicornia* [33,66,67,68].

Furthermore, our results indicated the necessity to address the possible adverse effect of ZnO NPs on living organisms.

### 4.1. ZnO NPs Reduce the Viability and Growth of BY-2 Cells

Our results imply that ZnO NPs in concentration 100 and 400 mg/L induced hypersensitive signals and a reduction of the growth of the BY-2 suspension. A decrease in the mitotic index indicated the inhibition of the cell division, which was involved in the reduction of growth parameters, PCV and fresh weight. The reduction of viability of ZnO NPs-treated BY-2 cells was more significant than the decrease in the growth. Our results corresponded to the ones described by Balažová, Lin and Xing, Kim et al., Dimkpa et al., i.e., who reported the ability of ZnO NPs to inhibit the growth of roots and shoots of several plant species, e.g., *Salicornia*, rape, ryegrass, wheat, cucumber and radish [33,69,70,71]. No effects (neither positive or negative) on growth were observed in BY-2 exposed to 10 mg/L ZnO NPs. Youssef et al. observed enhanced seed germination and seedlings growth of *Vicia faba* treated with 10 and 25 mg/L ZnO NPs, however, concentrations of 100 and 200 mg/L resulted in phytotoxicity [36]. The exact mechanism remains unknown. A suggested mechanism is based on the release of zinc ions from the ZnO NPs surface, i.e., a solubilization. Although zinc ions are essential for the plant growth, its reduction has been identified as a consequence of the interference of zinc ions released from ZnO NPs with activities of enzymes and uptake of nutrients [63]. An excess of zinc ions has been reported to be responsible for a biomass decline due to an inhibition of the cell elongation and division [72,73,74]. Upon the plant uptake (*Glycine max*, *Prosopis juliflora-velutina*) of Zn ions, their direct interaction with ribosomes and mitochondria, inducing the formation of reactive oxygen species (ROS), was observed [75,76,77]. In our case, observations employing a confocal microscopy confirmed increased concentrations of free zinc ions in BY-2 cells in a concentration-dependent manner. Zinc ions released from ZnO NPs were mainly observed in mitochondria.

### 4.2. The Fate of ZnO NPs and Their Effect on BY-2 Cells and Cellular Structures

Our study confirmed that ZnO NPs are able to release zinc ions during the cultivation. The same observation was found in case of *Salicornia* [33]. The highest solubilization was observed at the beginning of the experiment. Consequently, the ions release decreased with the time [69]. This fact was probably caused by the aggregation of ZnO NPs, which increased with the time. The aggregation and a subsequent solubilization processes were probably responsible for an earlier (24 h) effect of 100 mg/L ZnO NPs than that of 400 mg/L ZnO NPs. Furthermore, clustering of ZnO NPs could be responsible for a slower internalization of ZnO NPs in the highest concentration (400 mg/L). This process was connected with a decreased surface of ZnO NPs for a solubilization with clustering and creation of aggregates [78]. On the other hand, plant cells are able to synthesize and release many different compounds with different affinity to zinc ions. In addition, they are able to modify the pH value, which is crucial for the solubilization during the cultivation. It was shown that ZnO NPs are more toxic than Zn ions. For example in the case of *Allium cepa* plant, ZnO NPs showed stronger toxicity than zinc ions, even at concentrations lower than those necessary for ion toxicity [34,79]. In another study, Zn ions and ZnO NPs induced different morphological changes and different expression of metal homeostasis- and phytohormoneregulation-related genes in *Arabidopsis* seedlings [34,80]. In conclusion, this process is a very complex one and needs further investigation.

The second question connected with the fate of ZnO NPs is their internalization. Free zinc ions were traced in the cell wall of BY-2 cells after 24 h of the treatment in two highest concentrations, 100 mg/L and 400 mg/L, respectively. Later, they were observed inside BY-2 cells. The cell wall is a highly complex structure with 3–8 nm pores. Interactions between the cell wall and both ZnO NPs and free zinc ions can be expected. Study of Muschitz showed that almost 50% of zinc ions were confined into the cell wall [81]. In another study, the adverse effect on growth of eggplant seedling was based on accumulation of ZnO NPs (average particle size 35 nm) [68]. The transport of ZnO NPs through the cell wall is still discussed among scientists, but it seems that it is not limited by the size of pores. Some studies imply the ability of ZnO NPs to penetrate the cell wall [82,83], other indicate that the origin of ZnO NPs inside the cells is in free zinc ions released from NPs [84]. A much stronger signal of zinc ions in ZnO NPs variants indicates that BY-2 cells are capable of uptaking zinc ions released from ZnO NPs. On the other hand, we also have to consider a much stronger effect of ZnO NPs on BY-2 cells compared to zinc ions. Further study, which could confirm the transport of ZnO NPs through the cell wall and inside the plant cells, is necessary.

In our study, colocalizations of free zinc ions and Golgi apparatus/endoplasmic reticulum confirmed the crucial role of these organelles in the zinc homeostasis, which is in accordance with the work of Qin et al. [85]. The stress of endoplasmic reticulum probably plays one of the crucial roles in the ZnO NPs toxicity and may serve as an earlier biomarker for the NPs toxicity evaluation [86]. However, further studies of inductors and inhibitors of the ER stress are necessary, especially due to fact that all such studies were only performed on animal cell models [87]. Morphological changes in Golgi apparatus and endoplasmic reticulum observed during the ZnO NPs treatment may also help to clarify their obligatory role in the cell protection against high level of zinc ions. In addition, morphological changes in Golgi apparatus may be useful for further research, especially due to fact that zinc ions are necessary for the structural stability of a variety of proteins involved in the transcription and protein trafficking. Golgi apparatus also plays a crucial role in the maintaining zinc homeostasis by sequestrating excess ions, which helps maintain the proper cell functions [88,89].

### 4.3. ZnO NPs Increased the Level of ROS and RNS and Activated the ROS Defense System

The photocatalytic efficiency of ZnO promotes the production of ROS after irradiation [44], which leads to a significant toxicity [33,90,91]. In our case the BY-2 cells were cultivated in dark, so the effect of irradiation was negligible. Free zinc ions solubilized from ZnO NPs are able to catalytically stimulate the production of ROS by Fenton or Haber–Weissov reactions [92,93]. An increased level of ROS is regarded as one of the most important toxicological markers [40]. Therefore, we performed a histochemical visualization of ROS and measured the lipid peroxidation, activity of antioxidant enzymes and the amount of ROS scavengers, such as phenolics.

Confocal microscopy helped us to reveal the location of ROS and RNS in BY-2 cells and to co-localize them. The results of toxicity of Cd on *Zea mays* indicated that fluorescence microscopy is more sensitive to detect low change in oxidative balance (detection of H_2_O_2_ and superoxide) than the standard spectrophotometry [94].

Changes in the intensity of emission enabled us to quantify ROS and RNS. Surprisingly, nucleus with adjacent cytoplasm together with a plasma membrane lining the cell wall was one of the organelles with the highest amounts of ROS inside the cell. The presence of zinc ions in nucleus was confirmed in the highest concentration of ZnO NPs (400 mg/L). Therefore, despite the fact that zinc ions are able to regulate processes of transcription via different proteins, they may contribute to the creation of ROS [95].

Increased amounts of ROS affect activities (and production) of enzymes involved in the oxidation stress defense (e.g., SOD, PER, APER) [96]. A decreased activity of SOD, APER, and PER can be interpreted as their depletion resulting from the ROS overproduction. Effects of ZnO NPs on antioxidant enzymes activities were investigated in different plant species with several conclusions. Changes in APER similar to those in our study were reported by Cuypers et al. on a model plant *Phaseolus vulgaris* L. for ZnO NPs at the concentration of 50 mg/L [97] also on halophyte *Salicornia* at the concentration of 100 mg/L and 1000 mg/L ZnO NPs [33]. The activity of SOD decreased with increasing concentration (0, 3, 6, 12 mg/mL) of ZnO NPs in wheat callus cell, whereas the activity of PER increased [65]. ZnO NPs induced stronger depression of SOD and PER activities in wheat in comparison to ZnO bulk particles [37]. The depletion of catalase (CAT), SOD, and APER activities illustrate that the antioxidant defense system is “overloaded” with ROS. Opposite effects, the increase in CAT and APER activities, were demonstrated on a desert plant *Prosopis velutina* Woot. [75].

The ROS overproduction was followed by lipid peroxidation in all experimental variants of ZnO NPs. This indicates the incapacity of the antioxidant system to eliminate the excess of ROS. A free radical formation with an increased amount of malondialdehyde and a small amount of GSH were induced by zinc ions, as reported in [33,98,99,100]. The amount of malondialdehyde in safflower increased in a concentration-dependant manner after cultivation with 10, 100, 500, and 1000 mg/L ZnO NPs [101]. Thwala et al. (2013) suggested that the free radical activity in plant exposed to Ag NPs and ZnO NPs is linked to dissolved ionic species and also to a particulate form [100]. It has been shown that metal ion toxicity is based on the production of reactive oxygen species, but this is not the only mechanism of toxicity [102]. Based on the concentration of nanoparticles, the effects of both ionic and particulates form may synergistically lead to negative influence on the plants.

### 4.4. ZnO NPs Activate PAL and Enhance Amount of Metabolites with Scavenging Activity

Compounds with radical scavenging activity, such as phenolics and glutathione, are produced in plants exposed to stress conditions [56]. We observed the enhanced production of phenolics in our study. A significant increase in the total phenolics and flavonoids contents was observed in the study of Marichali et al., who exposed *Nigella sativa* L. to zinc ions [103]. Enhanced activity of PAL, which is an obligatory enzyme for production of phenolic compounds [56], was detected in the case of the BY-2 cells treated with the highest concentration of ZnO NPs (400 mg/L). The increased PAL activity leads to an increase in production and accumulation of phenolics. In addition, phenolics create an integral part of cell walls together with other biomolymers, such as polysaccharides. All of these compounds contribute to the detoxification of heavy metal ions and their immobilization in the cell wall. Cell wall represents an important target and barrier for heavy metal ions [104]. Increases in the antioxidative activities and non-enzymatic antioxidants (total phenolic and flavonoids) were found in the study of Zafar et al. The authors also found that ZnO NPs (500, 1000 and 1500 mg/L) adversely affect the *Brassica nigra* seed germination and seedling growth [105]. The overproduction of secondary metabolites with radical scavengers activity (mainly phenolics) was observed in the case of BY-2 cells treated with aluminium oxide NPs [46]. Enhanced activity of PAL is in agreement with the work of Kovacik et al., who reported similar changes in PAL activity on a model of *Matricaria chamomilla* L. In this case, the PAL activity was affected by nitrogen deficiency [56].

### 4.5. ZnO NPs Induce Processes of Autophagy

Subsequently, ROS may lead to epigenetic instability, for example through formation of 8-hydroxydeoxyguanosine. This fact has been confirmed on a model of human patients [106], however, no plant model has been applied until now. This is the first study on this topic. Epigenetic changes themselves may lead to PCD in both physiological [107] and non-physiological [108] manners. Despite the fact that connection between ROS and autophagy has been shown in many publications on animal cell models, knowledge about these connections in plants is rather small [109]. The presence of elevated amounts of zinc ions in cells leads to the production of ROS, which have ability to damage proteins, lipids, carbohydrates, and DNA. This ultimately results in a status called oxidative stress. Oxidative stress may lead to formation and accumulation of misfolded proteins [110]. As it has been shown on animal cell models, accumulated misfolded proteins are eliminated by degradation processes, such as autophagy [111]. In addition, the overproduction of ROS triggers signaling cascades leading to autophagy, which is required for the cellular adaptation to oxidative stress [112]. The primary step of autophagy is creation of small vacuoles called autophagosomes. This process is microscopically observed as a vacuolization [113]. In our case, it was observed in BY-2 cells upon the treatment with highest concentration of ZnO NPs (400 mg/L). The autophagosomes interact with lysosomes to create autolysosomes with high proteolytic activity [114]. Formation of autolysosomes is in correlation with increased activity of intracellular proteolysis [115]. A significantly higher protease activity in comparison with control was detected in the experimental variant of 400 mg/L already after 24 h. Formation of autophagosome membrane is contributed by different organelles, such as mitochondria [116], endoplasmic reticulum [117], or Golgi apparatus [118]. These facts were supported by microscopic observations of the morphological changes of the above-mentioned organelles in BY-2 cells treated with ZnO NPs. Vacuolization in root cortical cells was also observed in ryegrass [41] and *Allium cepa* after the exposure to ZnO NPs [119].

In our study, we observed aggregates of mitochondria in perinuclear region in BY-2 cell exposed to 400 mg/L ZnO NPs. Minority of mitochondrial aggregates was observed in cytoplasm adjacent to plasma membrane. The observations enable colocalization of these aggregates with places of creation ROS. Untreated BY-2 cells showed regular distribution of mitochondria in cytoplasm without signs of aggregation. Aggregates of mitochondria indicate their damage, which leads to their dysfunction. Impaired mitochondria, being a source of ROS, were observed mainly around nuclei in our study. Vacuolization of cells was well visible in the case of 400 mg/L ZnO NPs by using a probe for the Golgi apparatus. In addition, vacuolization was well evident using RNS and ROS probes. As was mentioned above, the connection between ROS and autophagy is often discussed in the literature and ROS are involved in it [120,121,122]. Zinc has shown an ability to regulate this process and the excess of zinc ions is involved in the initiation of processes of autophagy during oxidative stress [123]. One of the roles of autophagy is to degrade impaired organelles selectively. This quality-control autophagy in the case of mitochondria is called mitophagy. The intermediate requisite step of mitophagy is the production of mitochondrial aggregates [113]. Wei et al. (2017) that showed cytotoxicity induced by ZnO NPs leads to mitophagy [124]. We can conclude that ZnO NPs probably damage mitochondria and activate mitophagy, which was observed as the perinuclear mitochondrial aggregates.

### 4.6. ZnO NPs Induce Processes of PCD

Enzymes called caspases are responsible for regulation and execution of apoptosis in animal cells. However, enzymes with similar activity have also been found in plant cells. These enzymes are called caspase-like enzymes [125]. Metacaspases (cysteine-dependent proteases), saspases (subtilisin-like proteases), phytaspases (aspartate-specific proteases involved in plant cell death), and vacuolar processing enzymes are members of this group of enzymes responsible for programmed cell death, PCD in plant cells [126]. Using a substrate for caspase-3, we determined enhanced caspase-like activity in both concentration- and time-dependent manners in our work. The fragmentation of DNA usually occurs during PCD and it is one of the most important signs of PCD [39]. This phenomenon was investigated using TUNEL assay, which confirmed fragmentation of DNA, mainly in BY-2 cells exposed to 100 mg/L and 400 mg/L ZnO NPs. The fragmentation of DNA was accompanied by changes in nuclear architecture. Apoptotic-like, irregular and stretched nuclei, micronuclei, and nuclei with condensed chromatin were observed with increasing concentration of ZnO NPs. Genotoxicity of ZnO NPs and also TiO_2_ NPs were confirmed for *Allium cepa* root tips. Anaphase with multiple chromatin bridges and lagged metaphase were evidence of ZnO NPs and TiO_2_ NPs at concentrations as low as 0.1 mg/L [127]. Genotoxic effects of ZnO NPs on root cells of *Allium cepa* and *Vicia faba* were reported by Ghosh et al. [119]. ZnO NPs induced chromosomal aberration in *Vicia faba* in a concentration-dependent manner (10, 25 50, 100 and 200 mg/L) [36]. ZnO NPs showed stronger genotoxicity to wheat in comparison with bulk ZnO particles [37].

PCD should be also discussed from the viewpoint of cellular changes. They included mitochondrial aggregation and changes in ER structure. Mitochondrial aggregation is one of the signs of mitochondrial dysfunction and PCD. On the other hand, the role of mitochondrial aggregation around nuclei in plant cells remains almost unknown. However, a similar process has been described in animal cells. For example, Takada et al. have shown an ability of hepatitis B virus (HBV) X protein to induce creation of mitochondrial aggregates around nuclei of cells. In addition, the authors suggested that this event is closely connected with cell death [128]. It was shown that mitochondria-mediated apoptosis is associated with the toxicological effects of most nanomaterials on human cell and animal models [129]. Mitochondria play critical role in intrinsic apoptosis [27,130]. ZnO NPs cause mitochondria-mediated apoptosis in zebrafish embryos by reduction of the Bcl-2/Bax ratio and release of cytochrome C into the cytosol [129]. The connection between mitochondrial aggregation and apoptosis was shown in the work of Haga et al., who demonstrated that mitochondrial aggregation precedes release of cytochrome C1 from mitochondria during apoptosis [131]. In conclusion, mitochondrial aggregation may be taken into consideration as one of the signs of PCD.

The role of ER restructuring observed in our experiments remains unknown. There is only limited number of studies that focus this process. In addition, these studies are performed only on animal cell models. For example, Sprocati et al. induced ER restructuring by phenyl-2-decanoyl-amino-3-morpholino-1-propanol-hydrochloride. Effect of this compound was partially reversed by a membrane-permeable chelator of calcium ions. This fact indicates possible role of ER restructuring in processes of PCD. However, further studies on plant cells are needed. The results gave us evidence of induction of the PCD machinery by ZnO NPs in the BY-2 cells and provided another view on their phytotoxicity.

The genotoxicity of ZnO NPs wase also observed in the case of *Allium cepa* [79] and *Lathyrus sativus* [132]. In general, the genotoxicity followed by cytotoxicity occurs as a result of deregulation of components of the ROS-antioxidant machinery, and cell-cycle arrest, leading to DNA damage and, finally, cell death [119].

## 5. Conclusions

Nowadays, nanoparticles are widely used in different fields of industry and in medicine. However, data about their potential risk for the environment are still missing. Therefore, we focused on the effect of ZnO NPs (an example of the most common nanoparticles) on a model plant cell suspension culture of tobacco BY-2. Adverse effects of ZnO NPs on BY-2 cells were observed. It was found that ZnO NPs negatively affect the viability and growth of the cells. The observed effects correlated with the overproduction of ROS and initiation of processes of autophagy and programmed cell death. However, this study also revealed a couple of phenomena that need further clarification, (e.g., investigation of the ability of ZnO NPs to induce stress of endoplasmic reticulum, which plays a crucial role in handling and homeostasis of zinc ions. This process may be crucial for the initiation of both autophagy and PCD. In order to prove this, future studies are necessary. In general, the toxic effect of nanoparticles on the environment cannot be neglected and has to be seriously evaluated when considering their application.

## Figures and Tables

**Figure 5:**
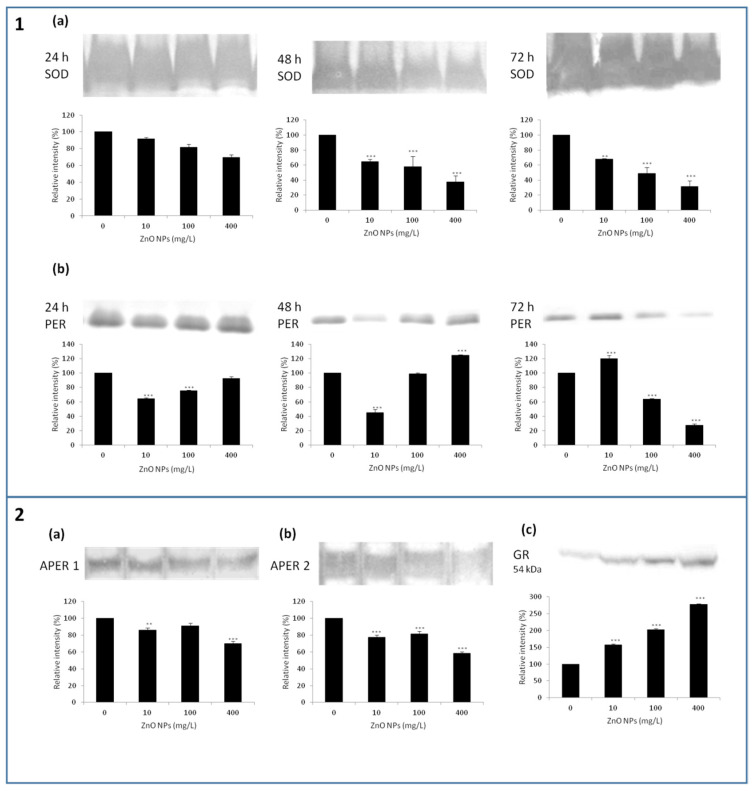
Time dependent response of tobacco BY-2 cells treated with ZnO NPs on superoxide dismutase, SOD (**1a**) and peroxidase, PER (**1b**) activities after 24, 48, and 72 h. The activity of two isoforms of ascorbate peroxidase, APER (**2a**,**b**) and dose-dependent response of BY-2 cells treated with ZnO NPs after 72 h on the amount of glutathione reductase, GR (**2c**) and. Each bar represents the mean of three independent experiments. ** indicates a significant difference between BY-2 cells treated with ZnO NPs and the corresponding control (untreated) BY-2 cells at *p* < 0.01, *** indicates a significant difference between the BY-2 cells treated with ZnO NPs and the corresponding control (untreated) BY-2 cells at *p* < 0.001. Bars represent standard deviations. All the results were normalized against protein concentrations of the cell lysates taken from the same samples.

**Figure 7:**
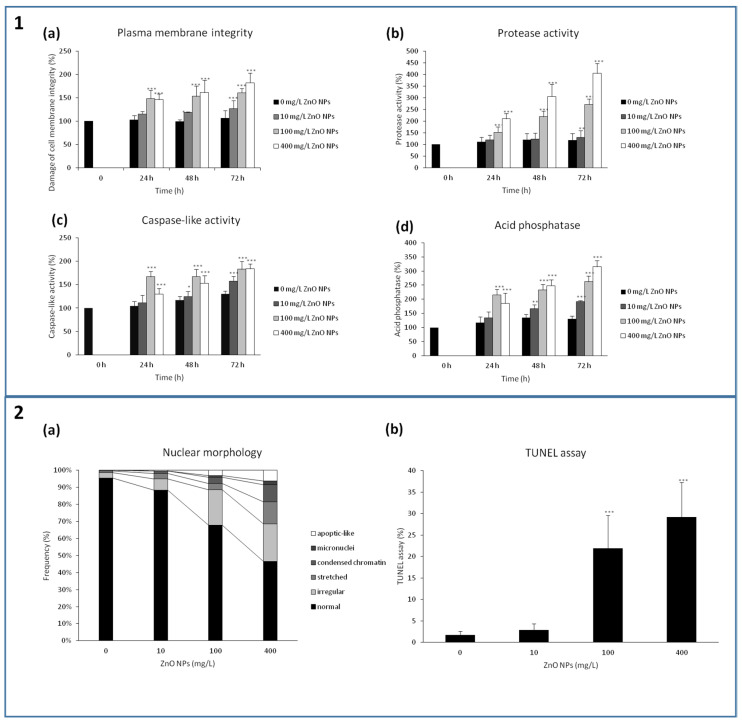
Effect of ZnO NPs on programmed death cell study by plasma membrane activity (**1a**), effect on autophagy evaluated by activity of proteases (**1b**), caspase-like activity (**1c**) and acid phosphatases (**1d**) after 72 h treatment with 0, 10, 100 and 400 mg/L ZnO NPs. Effect of ZnO NPs on nuclear cell morphology (**2a**) and DNA fragmentation (**2b**) after 72 h treatment with 0, 10, 100 and 400 mg/L ZnO NPs. * indicates a significant difference between the BY-2 cells treated with ZnO NPs and the corresponding control (untreated) BY-2 cells at *p* < 0.05, ** indicates a significant difference between the BY-2 cells treated with ZnO NPs and the corresponding control (untreated) BY-2 cells at *p* < 0.01, *** indicates a significant difference between the BY-2 cells treated with ZnO NPs and the corresponding control (untreated) BY-2 cells at *p* < 0.001. Bars represent standard deviations.
